# Exploring factors associated with breast cancer screening among women aged 15-49 years in Lesotho

**DOI:** 10.11604/pamj.2021.38.108.21110

**Published:** 2021-02-02

**Authors:** Kanono Thabane, Yolisa Mashologu, Lehana Thabane

**Affiliations:** 1 National University of Lesotho, Roma, Maseru, Lesotho,; 2 Lesotho Medical Dental and Pharmacy Council, Maseru, Lesotho,; 3 Department of Health Research Methods, Evidence, and Impact, McMaster University, Hamilton ON, Canada,; 4 Departments of Pediatrics and Anesthesia, McMaster University, Hamilton ON, Canada,; 5 Biostatistics Unit, St Joseph´s Healthcare, Hamilton, Hamilton ON, Canada

**Keywords:** Breast cancer, mammograpgy, mass screening

## Abstract

**Introduction:**

breast cancer is associated with serious morbidity, low quality of life and mortality. Prevention through early screening remains one of the most optimal strategies against breast cancer. The primary objective of this analysis was to determine the prevalence of breast cancer screening using the clinical breast examination (CBE) and breast self-examination (BSE) methods among women aged 15-49 years, and the secondary objective was to explore demographic and socio-economic factors associated with clinical breast examination (CBE) and breast self-examination (BSE) breast cancer screening methods.

**Methods:**

the study used Demographic Health Survey data collected in 2014. The study participants were Basotho women aged 15-49 years. STATA 17 was employed for developing logistic regressions and weighting for sampling probabilities and non-response. Complex sampling procedures were also considered during testing of statistical significance.

**Results:**

variables that were associated with significantly increased odds of having you had a breast cancer either self-examination or clinical test in last 12 months were: i) visiting a health centre in the past 12 months [odd ratio (OR): 1.21 (95% confidence interval [CI]: 1.02, p = 1.43); p = 0.025]; ii) completion of primary level education [1.27 ((1.10; 1.49); 0.001]; iii) being aware of breast cancer [2.18 (1.78;2.65); 0.001]; and iv) age [35-39 years: 1.40 (1.10;1.78);0.007]; while district of origin [Butha - Buthe: 0.63 (0.46; 0.85); 0.003] was significantly associated with decreased odds of the outcome.

**Conclusion:**

our findings suggest that raising awareness about breast cancer is the most effective method of improving breast cancer screening among women in Lesotho.

## Introduction

Breast cancer is associated with serious morbidity, low quality of life and mortality [1]. Treatment of breast cancer improves survival rate, but it is also associated with serious side effects including weight gain that leads to increased risk of developing obesity-related diseases, such as high blood pressure and diabetes [2]. Prevention through early diagnosis remains one of the most optimal strategies against breast cancer, and been shown to significantly improve outcomes by reducing morbidity and mortality [3]. Three methods are recommended for breast cancer screening, namely; (i) mammography, (ii) clinical breast examination (CSE) and (iii) breast self-examination (BSE) [4]. Mammography is defined as a technique using X-rays to diagnose and locate tumours of the breasts [5]. Clinical breast examination (CBE) is a physical exam done by a health professional [5]. Breast self-examination (BSE) is a technique which allows an individual to examine his/her breast tissue for any physical or visual changes [5]. Even though mammography is the most effective method for screening of breast cancer, its application in resource constrained developing countries has been unsustainable [6]. As a result, CSE and BSE are considered as alternative methods for screening breast cancer which could lead to early detection of breast cancer [6].

Several studies have investigated the factors associated with breast cancer screening. The first strand of studies investigated the factors associated with undertaking a mammography [6-14]. The second strand of studies have analysed factors associated with clinical breast examination (CBE) [15-18] while the third strand of studies analysed factors associated with breast self-examination (BSE) [17, 19-26]. Common socio-economic and demographic factors associated breast cancer screening in the form of CBE and BSE from literature were: (i) higher age [15, 19, 27-29]; (ii) higher awareness of breast cancer [16, 28-30]; (iii) higher education level [15, 31]; (iv) higher wealth [32]; (v) high perceptions on the importance of breast cancer screening [16, 33, 34]; and (vi) having access to health insurance [28, 31, 35]. Understanding these factors is of importance to low income countries such as Lesotho in order to develop policies and programmes which promote breast cancer screening and ultimately detection of breast cancer amongst women.

The primary objective of this study is to investigate the prevalence of breast screening among women aged 15-49 using CBE and BSE methods in Lesotho. The secondary aim, is to analyse the socio-economic and demographic factors which are associated with breast cancer screening, using CBE and BSE, among women aged 15-49 in Lesotho. Currently the prevalence of breast cancer screening is unknown for Lesotho due to absence of National Cancer Registry. Secondly, confirmed cases of breast cancer are not treated locally, they are treated in South Africa and India. In most cases, referrals are made where breast cancer is detection at an advanced stage. As a result, policy interventions which promote breast cancer screening are essential for developing countries such as Lesotho.

## Methods

### Study participants and setting

The study participants were Basotho women aged 15-49 years from the 10 districts of Lesotho in Southern Africa.

### Study design

This study is an exploratory analysis of secondary data derived from the 2014 national demographic health survey (DHS) database. DHS is designed to provide estimates of national and regional health indicators for the 10 districts of Lesotho. The sampling frame used for the 2014 LDHS is an updated frame from the 2006 Lesotho Population and Housing Census (PHC) provided by the Lesotho Bureau of Statistics (BOS). The sampling frame excluded nomadic and institutional populations such as persons in hotels, barracks, and prisons.

The 2014 LDHS followed a two-stage sample design and was intended to allow estimates of key indicators at the national level as well as in urban and rural areas, four ecological zones, and each of Lesotho´s 10 districts [36]. The first stage involved selecting sample points (clusters) consisting of enumeration areas (EAs) delineated for the 2006 PHC [36]. A total of 400 out 4000 clusters were selected, 118 in urban areas and 282 in rural areas. The second stage involved systematic sampling of households. A household listing operation was undertaken in all of the selected EAs in July 2014, and households to be included in the survey were randomly selected from these lists [36]. A total of 25 households were selected from each sample point, for a total sample size of 9,942 [36].

**Figure 1:**
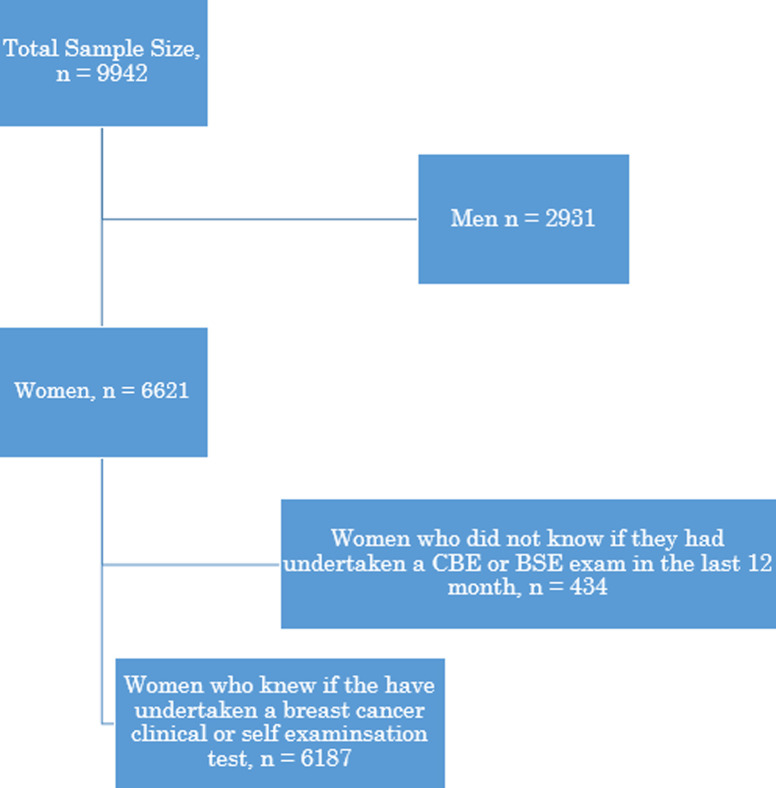
flow of respondents used in the analysis

We included all women aged 15-49 years for whom we had complete data on all the variables-which included the outcome and independent variables described below. As a result, out of 9,942 participants, there were 6621 women and 2931 were men. From a sample of 6,621 women, only 6187 were eligible for this study because they had knowledge of undertaking either a CBE or BSE in the last 12 months as shown in Figure 1. The other 434 (6621-6187) were not aware of whether they undertook a CBE or BSE in the last 12 months.

### Outcome variable

The outcome variable was: have you had a BSE or CBE in last 12 months? (yes/no)

### Independent variables

Age (in years), wealth index, district of origin, living in a rural or urban area, owning a radio or TV set, having a health insurance policy, awareness of breast cancer, visiting a health centre in the last 12 months, were among the independent variables.

### Statistical methods

We used descriptive statistics - reported as count (percentage) - to describe the characteristics of the respondents included in the analysis. We used a multivariable logistic regression to explore whether the independent factors where associated with the outcome. This analysis appropriately adjusted for the complex sampling design by using the design weights [37]. We also explored the potential interactions between awareness of breast cancer and the other independent factors. We assessed goodness-of-fit and model performance using the C-statistic. The results are reported as odds ratio [OR], corresponding 95% confidence interval [CI], and associated p-values. All p-values are reported to three decimal places with those less than 0.001 reported as p < 0.001. The criterion for statistical significance was set at alpha = 0.05. All analyses were performed using STATA 17.

## Results

### Key demographics

It is estimated that 3,887/6,187 (62.8%) of the study participants were form rural areas as shown in Table 1. In addition, it estimated that 4253/6187 (68.7%) of the study participants own a radio or TV while 3945/6187 (63.7%) of the participants have visited a health centre in the past 12 months. The results further indicated that 5381/6187 (87%) of the study participants were aware of breast cancer.

**Table 1:** demographic description of the sample

		Had a breast cancer self-examination or clinical test? n = 2385	Had no breast or clinical cancer Exam n = 3802	All n = 6187
Variable description	Category	n (%)	n(%)	n(%)
Do you live in a rural area?	Yes	1369 (22.1)	2518 (40.7)	3887 (62.8)
Do you own a radio or TV Set?	Yes	1708 (27.6)	2545 (41.1)	4253 (68.7)
Are you covered by health insurance?	Yes	64 (1.)	65 (1)	129 (2)
Have you visited a health centre in the past 12 months?	Yes	1623 (26.2)	2322 (37.5)	3945 (63.7)
Are you aware of breast cancer?	Yes	2220 (35.9)	3162 (51.1)	5381 (87)
Age (years)	15-19	442 (7.1)	892 (14.4)	1334 (21.5)
	20-24	467 (7.5)	744 (12.0)	1211 (19.5)
	25-29	432 (7)	581 (9.4)	1013 (16.4)
	30-34	352 (5.7)	549 (8.9)	901 (14.6)
	35-39	308 (5)	399 (6.5)	707 (11.5)
	40-49	384 (6.2)	638 (10.3)	1022 (16.5)
District of origin	Maseru	768 (12.4)	1011 (16.3)	1779 (28.7)
	Leribe	384 (6.2)	583 (9.4)	967 (15.6)
	Berea	333 (5.4)	489 (7.9)	822 (13.3)
	Butha Buthe	99 (1.6)	265 (4.2)	364 (5.8)
	Mafeteng	214 ( 3.5)	322 (5.2)	536 (8.7)
	Mohale's Hoek	176 (2.9)	303 (4.9)	479 (7.7)
	Quthing	102 (1.7)	185 (3)	287 (4.7)
	Qacha's Nek	74 (1.2)	123 (2)	197 (3.1)
	Thaba Tseka	107 (1.7)	218 (3.5)	325 (5.3)
	Mokhotlong	128 (2.1)	303 (4.9)	431 (7)
Wealth index	Poorest	278 (4.5)	618 (10)	896 (14.5)
	Poorer	319 (5.2)	637 (10.3)	956 (15.5)
	Middle	439 (7.1)	726 (11.7)	1165 (18.8)
	Rich	623 (10.1)	895 (14.5)	1518 (24.5)
	Richest	726 (11.7)	926 (15)	1652 (26.7)

### Key findings

**Prevalence of breast cancer screening:** it is estimated that 38.5% (2,382/6187) [95% CI: 36.6%, 40.2%] of the respondents had undertaken either a BSE or CBE in the past 12 months.

**Factors associated with breast cancer screening:** as shown in Table 2, variables that were associated with significantly increased odds of having either BSE or CBE in last 12 months were: i) visiting a health centre in the past 12 months [OR: 1.21 (95% CI: 1.02,1.43); p = 0.025]; ii) completion of primary level education [1.27 ((1.10; 1.49); 0.001]; iii) awareness of breast cancer [2.18 (1.78;2.65); 0.001]; and iv) women between age 35-39 years [1.40 (1.10;1.78);0.007].

**Table 2:** results of the multivariable logistic regression n = 6187

Variable		OR	95% Confidence interval	P-value	VIF
Do you live in a rural area	Yes	0.85	(0.71;1.04)	0.118	1.64
Do you own a radio or TV Set?	Yes	0.96	(0.82; 1.12)	0.607	1.40
Are you covered by health insurance?	Yes	1.14	(0.79; 1.66)	0.364	1.04
Have you visited a health centre in the past 12 months	Yes	1.21	( 1.02; 1.43)	0.025	1.11
Have you completed primary level education	Yes	1.27	(1.10; 1.49)	0.001	1.35
Are you aware of breast cancer?	Yes	2.18	(1.78;2.65)	0.001	1.16
Age (years)	15-19	Reference			
	20-24	1.13	(0.93;1.37)	0.219	1.55
	25-29	1.35	(1.07;1.68)	0.009	1.53
	30-34	1.14	(0.90;1.45)	0.273	1.50
	35-39	1.40	(1.10;1.78)	0.007	1.45
	40-49	1.12	(0.92;1.37)	0.244	1.61
District of origin	Maseru	Reference			
	Leribe	0.93	(0.73; 1.19)	0.583	1.64
	Berea	0.95	(0.72; 1.26)	0.741	1.61
	Butha Buthe	0.63	(0.46; 0.85)	0.003	1.56
	Mafeteng	0.97	(0.76; 1.23)	0.807	1.54
	Mohale's Hoek	0.92	(0.69; 1.22)	0.557	1.53
	Quthing	0.92	(0.71; 1.19)	0.511	1.50
	Qacha's Nek	0.97	(0. 73; 1.29)	0.844	1.55
	Thaba Tseka	0.85	(0.64; 1.13)	0.265	1.63
	Mokhotlong	0.75	(0.53; 1.17)	0.111	1.64
Wealth Index	Poorest	Reference			
	Poorer	0.98	(0.77; 1.25 )	0.871	1.74
	Middle	1.03	(0.80; 1.32)	0.810	2.13
	Rich	1.02	(0.86; 1.35)	0.864	2.85
	Richest	1.03	(0.86; 1.42)	0.858	3.81

OR: odds ratio; VIF: variance inflation factor;

Women from Butha Buthe district [Butha-Buthe: 0.63 (0.46; 0.85); 0.003] were significantly associated with decreased odds of the outcome relative to Maseru which is capital district of Lesotho.

There was no significant association between having either a BSE or CBE in last 12 months and owning a medical insurance. Similarly, the outcome variable was not significantly associated with being from a rural area, owning a radio or television set, household wealth and originating from other districts excluding the capital district which was used as a reference district.

The study also investigated the relationship between district of origin, breast cancer awareness and the outcome variable. The results of the main and interaction effects shown in Table 3 indicate that there is there was a significant interaction effect between breast cancer awareness and certain district of origin. The interaction effects reveal that women from Butha Buthe, Mokhotlong, Thaba Tseka, Mafeteng, Mohale´s Hoek and Quthing districts are most likely to undertake either a CBE or BSE if they were aware of breast cancer. In addition, the interaction effects reveal that women between ages 25-29 are likely to undertake either a CBE or BSE if they were aware of breast cancer.

**Table 3:** results of the multivariable logistic regression n = 6187

Variable		OR	95% Confidence interval	P-value	VIF
**Main effects**					
Do you live in a rural area?	Yes	0.86	(0.71: 1.04)	0.120	
Do you own a radio or TV Set?	Yes	0.96	(0.82; 1.13)	0.643	1.39
Do you have medical insurance?	Yes	1.13	(0.78; 1.64)	0.523	1.04
Have you visited a health centre in the past 12 months	Yes	1.21	(1.03; 1.44)	0.024	1.11
Are you aware of breast cancer?	Yes	4.34	(2.41; 7.80)	0.001	8.23
Have you completed primary level education	Yes	1.35	(1.17; 0 1.57)	0.001	1.33
Age (years)	15-19	Reference			
	20-24	1.14	(0.93; 1.38)	0.203	1.56
	25-29	1.36	(1.08; 1.70)	0.009	1.54
	30-34	1.14	(0.90; 1.45)	0.275	1.51
	35-39	1.40	(1.10; 1.78)	0.007	1.45
	40-49	1.12	(0.92; 1.37)	0.241	1.62
District of origin	Maseru	Reference			
	Leribe	0.62	(0.63; 3.51)	0.370	15.58
	Berea	0.48	(0.7; 5.01)	0.183	14.92
	Butha=Buthe	0.39	(0.62; 3.57)	0.379	17.19
	Mafeteng	0.34	(1.09; 6.46)	0.032	12.82
	Mohale's Hoek	0.43	(0.87; 4.69)	0.101	9.51
	Quthing	0.32	(1.20; 5.45)	0.015	7.89
	Qacha's Nek	0.58	(0.73; 3.80)	0.225	8.16
	Thaba Tseka	0.43	(0.87; 4.06)	0.106	8.72
	Mokhotlong	0.42	(0.75; 3.79)	0.208	7.84
Wealth Index	Poorest	Reference			
	Poorer	0.99	(0.78; 1.27)	0.959	1.75
	Middle	1.04	(0.81; 1.34)	0.734	2.07
	Rich	1.03	(0.78; 1.37)	0.818	2.52
	Richest	1.03	(0.75; 1.42)	0.840	2.96
Interaction effects: Breast Cancer Awareness*District of Origin*Outcome Variable					
District of Origin	Maseru	Reference			
	Leribe	0.62	(0.27; 1.42)	0.256	16.66
	Berea	0.48	(0.20; 1.18)	0.109	15.94
	Butha=Buthe	0.39	(0.16; 0.97)	0.043	18.50
	Mafeteng	0.34	(0.14; 0.86)	0.022	13.55
	Mohale's Hoek	0.43	(0.18; 1.01)	0.053	9.94
	Quthing	0.32	(0.15; 0.67)	0.003	8.08
	Qacha's Nek	0.58	(0.25; 1.31)	0.189	8.31
	Thaba Tseka	0.43	(0.20; 0.93)	0.032	9.01
	Mokhotlong	0.42	(0.20; 0.88)	0.021	7.91

OR: odds ratio; VIF: variance inflation factor

## Discussion

**Key findings:** based on the multivariable logistic regression estimated above, it is evident that having visited a health centre in the past 12 months, completing primary level education, awareness of breast cancer and age group 35-39 years were significantly associated with having you had either a BSE or CBE in last 12 months.

The results from the interaction effects reveal that lack of knowledge about breast cancer is the main challenge for women from Butha Buthe, Mokhotlong, Thaba Tseka, Mafeteng, Mohale´s Hoek and Quthing districts to undertake either a CBE or BSE.

**How results compare with similar studies:** these results are congruent with existing literature which indicates that breast cancer screening is highly associated with: (i) higher ager [15, 19, 27-29]; (ii) higher awareness of breast cancer [16, 28-30]; and (iii) higher education level [15, 31]; The results present a new variable, having visited a health centre, which is lacking from existing literature. In addition, the interaction between breast cancer knowledge, district of origin and the outcome variable provides a new an alternative method of analysing the association.

### Implications of the results

The results further indicate that having visited a health centre in the past 12 months, having completed primary level education, and being aware of the breast cancer, were associated with significantly higher the odds of having either a BSE or CBE in last 12 months.

The interaction results indicate the importance of breast cancer awareness on undertaking either a CBE or BSE. Firstly, the odds of women undertaking either a CBE or BSE are significantly increased amongst women with knowledge of breast cancer as compared to with women without knowledge of breast cancer. Secondly, women from certain districts of the country, namely Butha Buthe, Mokhotlong, Thaba Tseka, Mafeteng, Mohale´s Hoek and Quthing, are not undertaking either a CBE or BSE because they do not have knowledge of breast cancer. Thirdly, the results reveal that younger women can undertake breast cancer screening if they were aware of breast cancer. Lastly, the results reveal that awareness raising yields significant outcomes when undertaken at health centres as compared to radio or TV.

***Limitations:*** this is an exploratory analysis based on cross-sectional data and therefore we caution against inferring causal links in the results. Secondly, the study prevalence is based on self-reported data as opposed to clinical surveillance and we therefore caution its adoption as the national prevalence of breast cancer screening. Thirdly, the study focused on BSE and CBE as methods of breast cancer screening due to limited data on mammography.

## Conclusion

The prevalence rate of breast cancer screening, measured using BSE and CBE, among women aged 15-49 in Lesotho is estimated at 38.5%. In order to improve it, policy interventions should seek to improve knowledge of women on breast cancer. The interaction results indicate awareness campaigns are most effective when conducted at health centres as opposed to radios or television.

### What is known about this topic

- Breast cancer screening in developing countries can be measured using BSE and CBE;
- There are socio-economic factors affecting breast cancer screening, which are country and location specific;
- Media advocacy is one of the most effect tool for promoting breast cancer screening.

### What this study adds

- The study estimated the prevalence of breast cancer screening in Lesotho, measured using BSE and CBE, which was not known;
- The study presents new socio-economic variables which are associated with breast cancer screening; having visited a health centre in the last 12 months;
- The study proves that health education at health facilities on breast cancer is more effective than media advocacy in promoting breast cancer screening amongst women.
